# Vitamin C Mitigates Oxidative Stress and Tumor Necrosis Factor-Alpha in Severe Community-Acquired Pneumonia and LPS-Induced Macrophages

**DOI:** 10.1155/2014/426740

**Published:** 2014-09-01

**Authors:** Yuanyuan Chen, Guangyan Luo, Jiao Yuan, Yuanyuan Wang, Xiaoqiong Yang, Xiaoyun Wang, Guoping Li, Zhiguang Liu, Nanshan Zhong

**Affiliations:** ^1^Respiratory Section, Affiliated Hospital of Luzhou Medical College, Luzhou 646000, China; ^2^Hygiene Section, Luzhou Medical College, Luzhou, Sichuan 646000, China; ^3^State Key Laboratory of Respiratory Disease for Allergy at Shenzhen University, School of Medicine, Shenzhen University, Nanhai Avenue 3688, Shenzhen, Guangdong 518060, China; ^4^State Key Laboratory of Respiratory Disease, Guangzhou Medical University, Guangdong 510120, China

## Abstract

Oxidative stress is an important part of host innate immune response to foreign pathogens. However, the impact of vitamin C on oxidative stress and inflammation remains unclear in community-acquired pneumonia (CAP). We aimed to determine the effect of vitamin C on oxidative stress and inflammation. CAP patients were enrolled. Reactive oxygen species (ROS), DNA damage, superoxide dismutases (SOD) activity, tumor necrosis factor-alpha (TNF-*α*), and IL-6 were analyzed in CAP patients and LPS-stimulated macrophages cells. MH-S cells were transfected with RFP-LC3 plasmids. Autophagy was measured in LPS-stimulated macrophages cells. Severe CAP patients showed significantly increased ROS, DNA damage, TNF-*α*, and IL-6. SOD was significantly decreased in severe CAP. Vitamin C significantly decreased ROS, DNA damage, TNF-*α*, and IL-6. Vitamin C inhibited LPS-induced ROS, DNA damage, TNF-*α*, IL-6, and p38 in macrophages cells. Vitamin C inhibited autophagy in LPS-induced macrophages cells. These findings indicated that severe CAP exhibited significantly increased oxidative stress, DNA damage, and proinflammatory mediator. Vitamin C mitigated oxidative stress and proinflammatory mediator suggesting a possible mechanism for vitamin C in severe CAP.

## 1. Introduction

Oxidative stress is a key part of the chain of events leading to inflammation caused by bacterial infection. Granulocyte peroxidases play an important role in triggering oxidative stress [1]. Cystic fibrosis (CF) has been associated with oxidative stress, in particular during the chronic pulmonary infection with* Pseudomonas aeruginosa*, which is the main cause of morbidity and mortality in CF [2]. Respiratory syncytial virus (RSV) infection caused oxidative cell damage and cellular signaling in modulating virus-induced lung disease [3]. Reactive oxygen species (ROS) and oxidative stress are thought to play a central role in the etiology of cell dysfunction and tissue damage. ROS also modulate a number of cell signaling pathways resulting in transcription factor activation and inflammatory mediators' release [4]. ROS induce vascular cell adhesion molecule-1 (VCAM-1) signal transduction and VCAM-1-dependent inflammation is blocked by antioxidants [5]. A study reported the occurrence of higher oxidative stress in bacterial severe community-acquired pneumonia patients [6]. Antioxidants may affect pulmonary morbidity. Vitamin C significantly improved the “total respiratory score” in the most severely ill patients [7].

Lipopolysaccharide (LPS; endotoxin) is an important event that contributes to the elevation in reactive oxygen species [8]. LPS-induced acute lung injury (ALI) and aberrant proliferation of lung fibroblasts initiated in early disease stages are associated with PI3K-Akt pathway activation [9]. ROS induced DNA damage. Spontaneously, endogenous DNA damage can activate NF*κ*B. Inhibiting the canonical NF-*κ*B pathway exacerbated H_2_O_2_-induced A549 cell apoptosis [10]. Autophagic cell death plays a crucial role in infection. H5N1-infected lungs from a human cadaver, mice, and infected A549 human epithelial lung cells show the accumulation of autophagosomes. Blocked autophagic signaling increased the survival rate of mice and meliorated the acute lung injury and mortality caused by H5N1 infection [11]. Oxidative stress-induced DNA damage and autophagy remain unclear in pneumonia. In our present study, our findings indicated that severe CAP exhibited significantly increased oxidative stress and proinflammatory mediator. Vitamin C mitigated oxidative stress and proinflammatory mediator.

## 2. Material and Methods

### 2.1. Study Design and Subjects

The study was conducted at affiliated hospital of Luzhou medical college (a 3000-bed hospital in Luzhou City, Sichuan, China). All patients admitted to the hospital with pneumonia between July 2011 and June 2013 were recruited. Pneumonia was defined as a new infiltrate in chest radiography together with symptoms and signs of a lower respiratory tract infection: fever (>38°C), cough, and purulent sputum [12, 13]. Multiple severity scoring systems have been devised and evaluated in community-acquired pneumonia. Patients with pneumonia were classified into mild to moderate pneumonia (no severe pneumonia) and severe pneumonia according to the primary care summary of the British Thoracic Society Guidelines for the management of community-acquired pneumonia (CAP) in adults [14]. Patients who have a CRB-65 score of 3 or 4 were defined as severe pneumonia. This study was approved by the Luzhou Medical College Ethic Committee.

### 2.2. Mononuclear Cell Separation and Human Lung Tissue

Peripheral blood obtained from pneumonia and normal donor was processed for separation of mononuclear cells. Peripheral blood was diluted 1 : 1 with sterile phosphate-buffered saline (PBS), layered over Ficoll-Hypaque (GE Healthcare Bio-Sciences AB, Stockholm, Sweden), and centrifuged at 1500 rpm for 15 min at room temperature. Peripheral blood mononuclear cells (PBMC) were collected from the interphase layer and washed with PBS. PBMC were resuspended with RPMI-1640 medium. Cells were grown in RPMI-1640 medium containing 10% FCS (fetal calf serum). The lung tissues were collected by lung tissue biopsy with fiber bronchoscope. Tissues were quickly immersed in liquid nitrogen and transferred to −70°C refrigerator frozen storage.

### 2.3. MH-C Cells Culture

When murine alveolar macrophage* cell* lines (MH-C cells) were grown to 85% confluence in RPMI-1640 medium containing 10% FCS, the medium was replaced with serum-free RPMI-1640 culture medium. The cells were then treated with LPS or/and 100 nM vitamin C in serum-free culture medium for 24 hours.

### 2.4. Measurement of ROS, SOD, IL-6, and TNF-*α*

The cells were loaded with 10 *μ*M H2DCF-DA (Invitrogen, Molecular Probes, USA) or 10 *μ*M dihydroethidium (DHE, Invitrogen, Molecular Probes, USA) at 37°C for 30 minutes according to the manufacturer's instructions. After removing excess probes, the cells were kept at 37°C containing 5% CO_2_. Fluorescence intensity was detected by Leica TCS SP5 confocal microscope (Leica, Germany). For each sample 10,000 events were collected. For lung tissues, add 0.1% ROS (DHE) probe about 10 edged up on the lung tissue of frozen section, after incubation for 30 minutes at 37°C, washing 2-3 times with phosphate-buffered saline (PBS) and observation by Leica TCS SP5 confocal microscope. Superoxide dismutases (SOD) activity was measured using the SOD assay kit WST (Nanjing jiancheng bioengineering institute, china). The ELISA kits of TNF-*α* and IL-6 were purchased from R&D Systems (Minneapolis, MN).

### 2.5. Measurement of DNA Damage and Cell Viability

The comet assay was used to measure DNA damage. The procedure of comet assay was performed [15]. 10 *μ*L cells suspension containing 20,000 cells was mixed with 90 *μ*L low-melting-point agarose (LMA) (Sigma) in PBS at 37°C and was layered onto slides which had been coated with normal melting point agarose. The slides were submersed in freshly prepared cold (4°C) lysing solution (2.5 M NaCl, 100 mM EDTA-2Na, 10 mM Tris-HCl, pH 10–10.5, 1% Triton X-100, and 10% DMSO) for 2 hours. Slides were immersed in fresh electrophoresis buffer at 4°C for 30 min and then electrophoresed (25 V/300 Ma) for 25 min. After electrophoresis, the slides were stained with ethidium bromide. Slides were covered with a coverslip and analyzed using Leica TCS SP5 confocal microscope (Leica, Germany). Comet assay IV software was used to assess the DNA damage score. Cell viability was measured by 5-ethynyl-2′-deoxyuridine (EdU) assay using an EdU assay kit (Ribobio, Guangzhou, China) [16]. The cell nucleus was stained with 4′-6-diamidino-2-phenylindole (*DAPI*) reagents.

### 2.6. Cell Transfection

MH-S cells were transfected with RFP-LC3 or GFP-LC3 plasmids using Lipofectamine 2000 reagent (Invitrogen) in serum-free RPMI 1640 medium (Thermo Fisher Scientific) following the manufacturer's instructions [17]. RFP-LC3 and GFP-LC3 plasmids were kindly provided by Min Wu (the University of North Dakota, US).

### 2.7. Western Blot

Cells were homogenized in RIPA lysis buffer for western blot analysis. Lysates (20 *μ*g) were run on 10% SDS polyacrylamide gel at 100 V for 2 hours and transferred to microporous polyvinylidene difluoride (PVDF) membrane at 100 mA for 2 hours. The membrane was blotted with phosphorylated (p)-P38, P38, TNF-*α*, LC3, BECN1, and *β*-actin antibodies (1 : 1000) (Santa Cruz Biotechnology, Inc.) and processed via enhanced chemiluminescence (Pierce).

### 2.8. Statistical Analysis

Data are expressed as mean ± standard error. Statistical analysis was performed using ANOVA (Tukey's post hoc) or Student's *t*-test and the level of significance was defined as *P* < 0.05 between any two groups. Mann-Whitney *U* test and Spearman's correlation with a two-tailed test were used for statistical analyses. The data were analyzed using SPSS 13.0 software [18].

## 3. Results

### 3.1. Description of Severe CAP

A total of 30 patients with community-acquired pneumonia were enrolled in the study. 15 patients had severe community-acquired pneumonia and 15 patients with community-acquired pneumonia showed nonsevere pneumonia. 15 cases were admitted to control cases. The baseline characteristics of these patients are described in Table 1. The mean age of severe community-acquired pneumonia or nonsevere community-acquired pneumonia was not significantly different with that in normal control. Severe community-acquired pneumonia shown decreased PaO_2_ and PaO_2_/FiO_2_ compared to nonsevere community-acquired pneumonia (*P* = 0.001). The sputum culture showed the growth of* Klebsiella pneumonia*,* Escherichia coli*, and Acinetobacter. Endotoxins in severe CAP were significantly increased compared to nonsevere CAP.

### 3.2. Severe CAP Enhances Oxidative Stress in Lung

**Figure 1:**
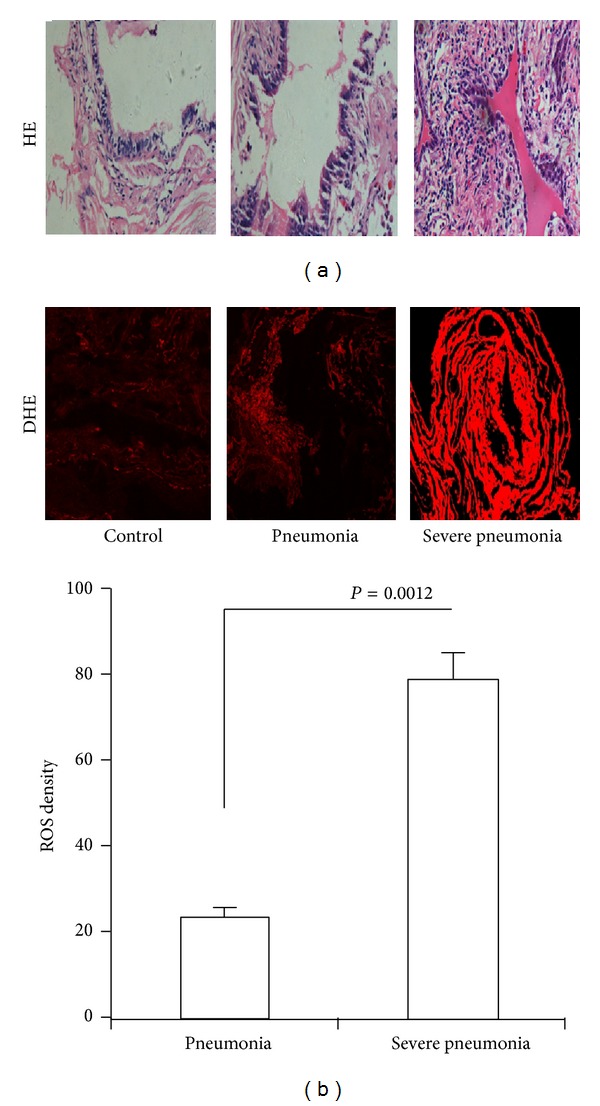
*Oxidative stress in severe CAP*. (a) HE staining of lung tissue from no severe CAP and severe CAP patients. (b) ROS were detected by confocal microscope using DHE probe. ROS level was represented by fluorescence intensity (×200).

Community-acquired pneumonia had higher oxidative stress compared with patients without infection [19]. However, it remains unclear that oxidative stress involves severe degree of pneumonia. To evaluate the oxidative stress of lung in CAP, we measured the ROS in airway tissue from control groups or from CAP patients collected by bronchoscopy. CAP patients exhibited increased ROS in airway tissue. ROS level of severe CAP significantly increased compared with that of nonsevere CAP (*P* = 0.0012, Figure 1(b)).

### 3.3. Severe CAP Enhances ROS, TNF-*α*, and IL-6

**Figure 2:**
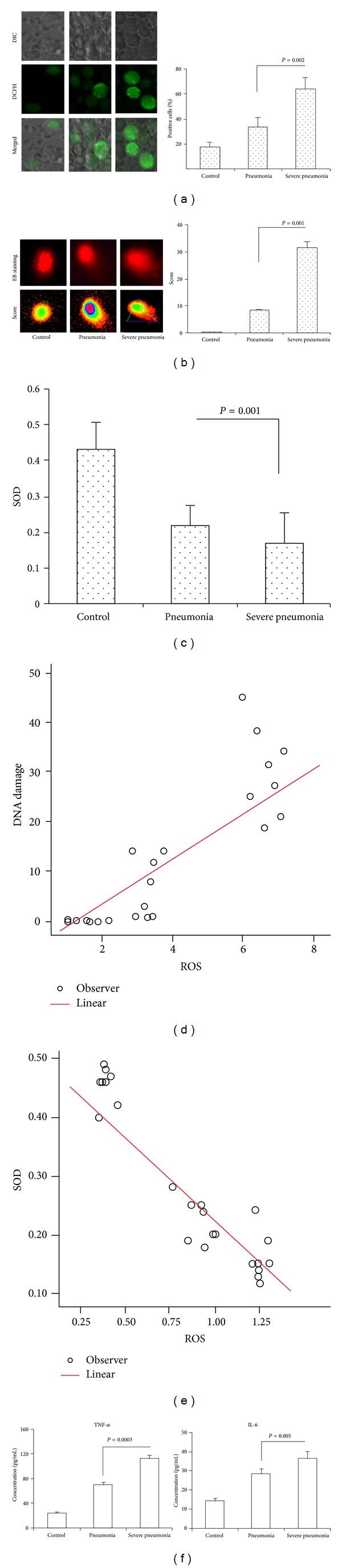
*Oxidative stress, DNA damage, TNF-*α*, and IL-6 in severe CAP*. (a) Intracellular ROS were detected by confocal microscope using DHE probe. ROS level was represented by fluorescence intensity (×400). (b) DNA damage was detected by comet assay using confocal microscope (×400). DNA damage score was analyzed with comet assay IV software. (c) SOD activity in serum was measured using the SOD assay kit WST. (d) Correlations between ROS and DNA damage were also evaluated. (e) Correlations between ROS and SOD were also evaluated. Spearman's correlation with a two-tailed test was used for statistical analyses. (f) Standard ELISA was performed to determine the levels of TNF-*α* and IL-6.

It has been shown that protection against postinfluenza bacterial pneumonia is by increasing phagocyte recruitment and ROS production [20]. However, whether excessive ROS aggravated inflammation remains unclear. Using ROS probe, we found that CAP showed increased ROS compared to control in PBMC. Furthermore, severe CAP showed increased ROS density compared to nonsevere CAP (*P* = 0.002, Figure 2(a)). DNA damage in PBMC was detected by comet assay. DNA damage of PBMC exhibited different increases in CAP. A total damage score for each slide in severe CAP was significantly increased compared to nonsevere CAP (*P* = 0.001, Figure 2(b)). The SOD in severe CAP was significantly decreased compared to nonsevere CAP (*P* = 0.001, Figure 2(c)). The significant association was found between ROS and DNA damage in severe CAP (*r* = 0.632, *P* = 0.007, Figure 2(d)). There is significant negative correlation between SOD and ROS (*r* = 0.632, *P* = 0.007, Figure 2(e)). The TNF-*α* and IL-6 in severe CAP were significantly increased compared to nonsevere CAP (*P* = 0.0003 and 0.005, Figure 2(f)). The results indicated that severe CAP had higher oxidative stress, DNA damage, and proinflammatory mediator production.

### 3.4. Vitamin C Decreases ROS, TNF-*α*, and IL-6 in Severe CAP

**Figure 3:**
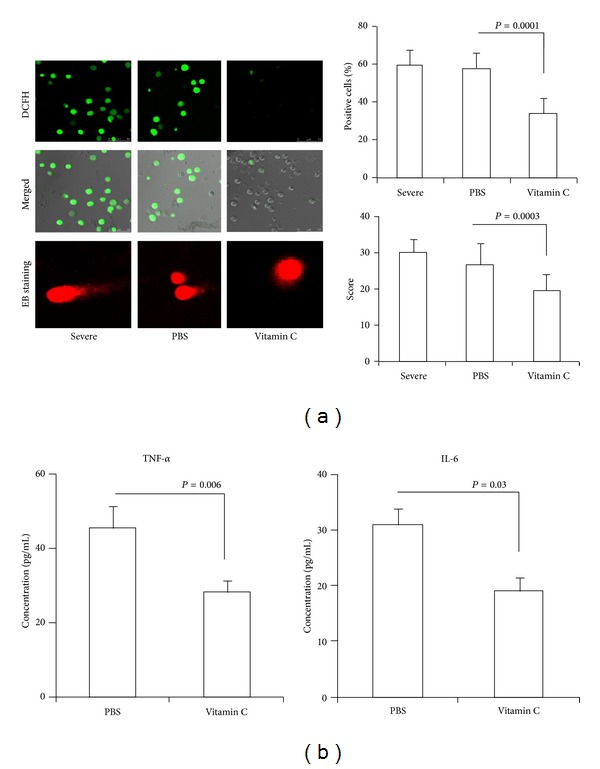
*The effect of vitamin C on ROS, DNA damage, TNF-*α*, and IL-6 in vitro*. Monocytes from severe CAP were treated with vitamin C for 12 h in vitro. (a) Intracellular ROS were detected by confocal microscope using DHE probe (×400). DNA damage was detected by comet assay using confocal microscope (×400). (b) Whole blood cells from severe CAP were treated with vitamin C for 12 h in vitro. Standard ELISA was performed to determine the levels of TNF-*α* and IL-6.

Vitamin C effectively inhibited amfepramone-induced DNA damage [21], but whether vitamin C inhibited bacterial infection and induced ROS and DNA damage in severe CAP remains unclear. Our studies found that vitamin C decreased ROS and DNA damage scores compared with PBS-treated monocytes from severe CAP in vitro (*P* = 0.0001 and 0.00053, Figure 3(a)). Vitamin C decreased TNF-*α* and IL-6 compared with PBS-treated whole blood cells from severe CAP in vitro (*P* = 0.006 and 0.03, Figure 3(b)).

### 3.5. Vitamin C Inhibited LPS-Induced ROS, TNF-*α*, and P38 in MH-S Cell Lines

**Figure 4:**
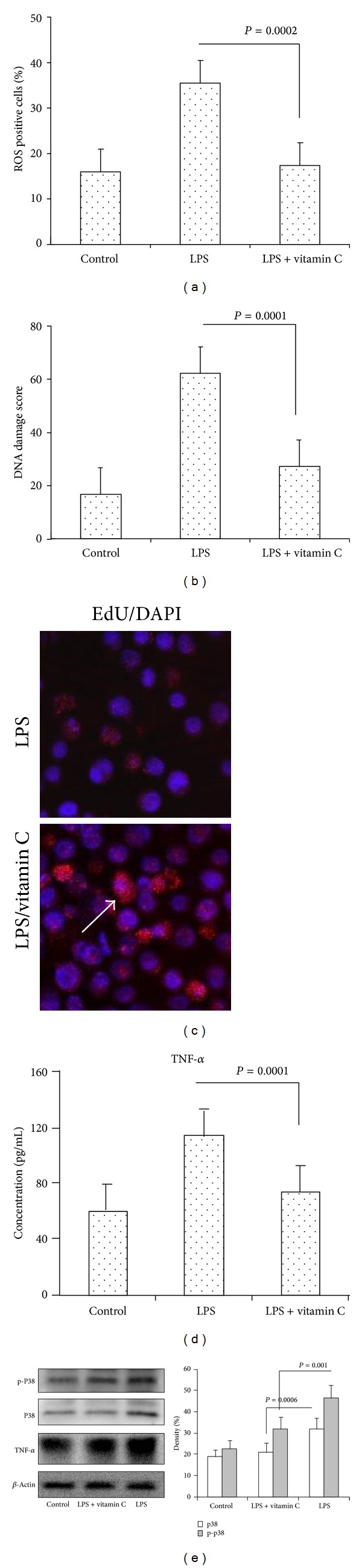
*The effect of vitamin C on oxidative stress and DNA damage in LPS-induced MH-S cells*. ROS and DNA damage in MH-S cells were measured in the presence or absence of vitamin C and treated with LPS for 12 hours. (a) ROS were detected by confocal microscope using DCFH-DA probe. ROS level was represented by fluorescence intensity. (b) DNA damage was measured by comet assay. DNA damage score was analyzed with comet assay IV software. (c) MH-S cells viability was detected by confocal microscope using Edu staining (×200). Arrow showed viability of cells. (d) TNF-*α* was measured by ELISA. (e) TNF-*α*, P38, and p-P38 were determined by western blotting. *β*-actin was used as the loading control.

Reactive oxygen species produced during the innate immune response to LPS are important agents of antipathogen defense, but whether vitamin C regulates LPS-induced oxidative stress and proinflammatory mediators remains unclear [22]. In present studies, MH-S cells were stimulated with LPS for 24 hours and vitamin C was added. We found that LPS increased ROS-positive cells and DNA damage score compared to control cells. Vitamin C inhibited ROS-positive cells and DNA damage score compared to LPS-stimulated cells (Figures 4(a) and 4(b), *P* = 0.0002 and 0.0001). Vitamin C increased MH-S cell viability in LPS-stimulated cells (Figure 4(c)). It has been shown that concentrations of TNF-*α* released from LPS-stimulated cells increased significantly [23]. Hydrogen peroxide induced TNF-*α* production in macrophages via activating p38 as oxidative stress-related signal pathways [24]. After vitamin C treatment, a significant decrease in TNF-*α*, P38, and p-P38 in LPS-stimulated cells was observed in present study (Figure 4(e), *P* = 0.001 and 0.0006).

### 3.6. Vitamin C Decreased LPS-Induced Autophagy in MH-S Cell Lines

**Figure 5:**
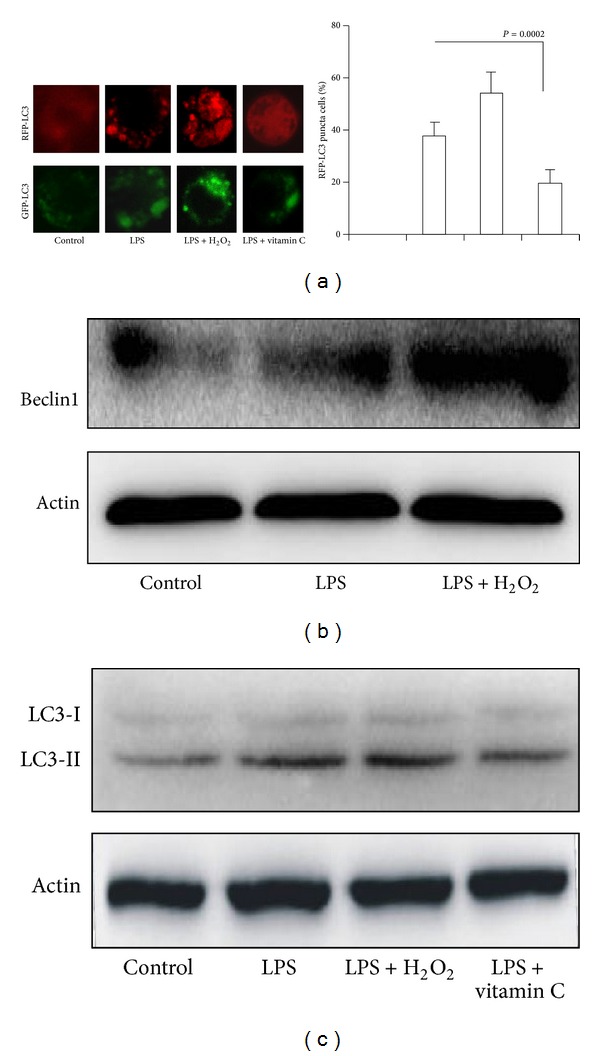
*The effect of vitamin C on LPS-induced autophagy in macrophages*. (a) MH-S cells were transfected with RFP-LC3 or GFP-LC3 plasmids for 24 hours. MH-S cells were cultured in the presence or absence of vitamin C and treated with LPS for 12 hours. (b) Western blotting of Beclin1; (c) western blotting of LC3. *β*-actin was probed as a loading control in (b) and (c).

It has been found that autophagy is required for an effective immune response against infection in vivo and enhances bacterial clearance during* Pseudomonas aeruginosa* lung infection [25]. LPS-induced autophagy is involved in the restriction of* Escherichia coli* in peritoneal mesothelial cells [26]. To determine whether LPS induces autophagy, MH-S cells were transfected with RFP-LC3 plasmids or GFP-LC3 plasmid. MH-S cells were stimulated with 10 *μ*g/mL LPS for 12 h. According to previous report, the confocal microscopy images were used to semiquantitatively measure [17]. We observed that LPS induced LC3 punctation in the MH-S cells. H_2_O_2_ significantly increased LC3 punctation. However, vitamin C inhibited the increased LC3 punctation in LPS-induced cells (Figure 5(a)). We found that H_2_O_2_ significantly increased beclin-1 in LPS-stimulated cells (Figure 5(b)). The expression of LC3-II was increased in LPS-stimulated cells. Vitamin C inhibited increased LC3II in LPS-stimulated cells (Figure 5(c)).

## 4. Discussion

Pneumonia is an infection of the lungs usually caused by bacteria and viruses [27]. Oxidative stress is an important part of host innate immune response to foreign pathogens [28]. Oxidative stress in the respiratory system increases the production of mediators of pulmonary inflammation. Such effects include increased expression of intercellular adhesion molecule 1 and interleukin-6 and hypersecretion of mucus [29]. Our study found that CAP exhibited increased ROS in lung tissue. In addition, severe CAP showed significantly increased ROS in lung. Therefore, these data indicated that oxidative stress involves severe degree of pneumonia.

It has been shown that respiratory syncytial virus infection induces significant downregulation of the airway antioxidant system in vivo, likely resulting in lung oxidative damage [30]. Antioxidants have been shown to be effective in preventing lung injury and protect against damage of other organs, such as heart, kidney, and liver in animal models of oxidative stress [31]. We found that ROS also increased in CAP PBMC. ROS significantly increased in severe CAP compared to CAP. Furthermore, CAP and severe CAP exhibited DNA damage in PBMC. Severe CAP exhibited more DNA damage compared to CAP. Oxidative damage is correlated with superoxide dismutase (SOD) in the lung. Antioxidant treatment reverses organ failure in rat model of sepsis [31]. In our present study, we found that SOD was negatively correlated with ROS in severe CAP PBMC. Severe CAP exhibited more DNA damage in PBMC. The TNF-*α* and IL-6 in severe CAP were significantly increased. The results indicated that oxidative stress and DNA damage likely represent an important pathogenetic mechanism of severe CAP.

The prophylactic use of vitamin C to prevent pneumonia should be further investigated in populations who have a high incidence of pneumonia [27]. Although it has been shown that vitamin C significantly improved the “total respiratory score” in the most severely ill patients, antioxidants may affect pulmonary morbidity. More research on vitamin C and other antioxidants seems to be warranted [7]. Antioxidants preserve macrophage phagocytosis of* Pseudomonas aeruginosa* during hyperoxia [32].

Our study is the first to report that vitamin C decreased ROS and DNA damage of severe CAP PBMC in vitro, and vitamin C decreased TNF-*α* and IL-6 in whole blood cells from severe CAP.

Reactive oxygen species (ROS) regulated inflammatory responses through the NF-*κ*B pathway [33]. Oxidative stress may alter the balance between gene expression of proinflammatory mediators and antioxidant enzymes in favor of inflammatory mediators in the lung [34]. LPS (ligand of TLR4) induced tumor necrosis factor-*α* (TNF-*α*) production in macrophage lines. LPS-induced TNF-*α* may be a useful therapeutic candidate for the treatment of sepsis and other inflammatory diseases [35]. p38 activation were associated with LPS-induced TNF-*α* in macrophage [36]. N-acetylcysteine significantly improved zymosan-induced lung tissue damage and impaired lung function [37]. However, antioxidants increased the severity of peritonitis by decreasing the phagocytic efficiency, oxidative burst, and TNF-*α* production and increasing neutrophil infiltration. Antioxidants reduced the phagocytic efficacy of peritoneal macrophages and also decreased* E. coli*-induced oxidative burst in macrophages cells. Antioxidant supplementation during the course of bacterial infection is not recommended as it could be detrimental for the host [38]. Vitamin C is a novel regulator of neutrophil extracellular trap formation in sepsis. Vitamin C is protective in sepsis settings [39]. Our data indicated that LPS induced increase of ROS and DNA damage in macrophage cell lines. The expressions of TNF-*α*, p38, and of phosphorylation p38 were also increased in LPS-stimulated macrophages cells. Vitamin C reduced the ROS level and DNA damage degree and also decreased expressions of TNF-*α*, p38, and phosphorylation p38 in LPS-stimulated macrophages cells in vitro.

Autophagy pathway is activated under environmental stress conditions [40]. The previously reported autophagy in vivo effectively regulates bacterial clearance of* P. aeruginosa* from the lung. Therapeutic intervention aimed at inducing autophagy with rapamycin correlates with decreased bacterial loads following* P. aeruginosa* lung infection in vivo [25]. LPS upregulates autophagy in hepatocytes; LC3II expression increased in both liver and hepatocytes after LPS and was dependent on TLR4 [41]. Indeed, our data convincingly showed that LC3 punctation increased in LPS-stimulated MH-S cells. H_2_O_2_ significantly increased LC3 punctation in LPS-stimulated MH-S cells. LC3 are widespread in the cells of various tissues, mainly expressed in autophagy body. LC3II expression increased in MH-S cells exposed to LPS and H_2_O_2_. Beclin1 expression increased in MH-S cells exposed to LPS and H_2_O_2_. The data indicated that oxidative stress unregulated autophagy, which might be useful for bacterial clearance. However, we found that vitamin C inhibited autophagy in MH-S cells exposed to LPS and H_2_O_2_. The effect of vitamin C on autophagy needs to be investigated.

In summary, we demonstrate that severe CAP exhibited significant increase of oxidative stress and proinflammatory mediators (TNF-*α* and IL-6) in lung and peripheral blood. Vitamin C inhibited ROS, DNA damage, TNF-*α*, and IL-6 from severe CAP in vitro. Vitamin C also reduced the ROS, DNA damage, and TNF-*α* production in LPS-stimulated macrophages cells. Oxidative stress unregulated LPS-induced autophagy in macrophages cells. Vitamin C inhibited autophagy in MH-S cells exposed to LPS and H_2_O_2_. Thus, our studies represent a novel mechanism of vitamin C by which it inhibited oxidative stress and proinflammatory mediators in severe pneumonia.

## Figures and Tables

**Table 1:** Descriptive statistical analysis of the study groups.

	Control	Nonsevere pneumonia	Severe pneumonia
Number	15	15	15
Sex, m/f	8/7	9/6	11/4
Age, years	64.28 (4.12)	61.26 (3.19)	65.21 (4.56)
PaO_2_ mmHg	—	99.8 (12.3)	41.2 (8.2)
PaCO_2_ mmHg	—	35.2 (4.8)	43.2 (3.9)
PaO_2_/FiO_2_ mmHg	—	321 (35)	196 (42)
*Klebsiella pneumoniae *	—	2	2
*Escherichia coli *	—	3	2
Cinetobacter	—	3	4
Endotoxin (pg/mL)	—	28.13 (4.14)	39.34 (5.12)

Data are shown as means (SD).

M: male; F: female; FiO_2_: fraction of inspired oxygen; ST: patient temperature during sampling.

## Abbreviations

ROS: Reactive oxygen species
LPS: Lipopolysaccharide
CAP: Community-acquired pneumonia
PBMC: Peripheral blood mononuclear cells
DHE: Dihydroethidium.
